# Early Dynamic Changes in Haematoma Thickness in Medically Managed Type A Intramural Haematoma: A Multicentre Retrospective Study

**DOI:** 10.1093/icvts/ivag146

**Published:** 2026-06-08

**Authors:** Yusuke Motoji, Tadashi Kitamura, Noritsugu Naito, Fumiaki Shikata, Yujiro Miura, Masaomi Fukuzumi, Hiroshi Okada, Hiroshi Matsushita, Tomoki Tamura, Tetsuya Horai, Kagami Miyaji, the Society for Promotion and Enhancement of Cardiovascular Surgery Group, Yusuke Motoji, Tadashi Kitamura, Noritsugu Naito, Fumiaki Shikata, Yujiro Miura, Masaomi Fukuzumi, Hiroshi Okada, Hiroshi Matsushita, Tomoki Tamura, Tetsuya Horai, Kagami Miyaji

**Affiliations:** Department of Cardiovascular Surgery, Kitasato University School of Medicine, 1-15-1 Kitasato, Minami-ku, Sagamihara-shi, Kanagawa, 252-0374, Japan; Department of Cardiovascular Surgery, National Center for Global Health and Medicine, 1-21-1 Toyama, Shinjuku-ku, Tokyo, 162-8655, Japan; Department of Cardiovascular Surgery, Jichi Medical University, 3311-1 Yakushiji, Shimotsuke-shi, Tochigi, 329-0498, Japan; Department of Cardiovascular Surgery, National Hospital Organization Shizuoka Medical Center, 762-1 Nagasawa, Shimizu-cho, Sunto-gun, Shizuoka, 411-8611, Japan; Department of Cardiovascular Surgery, Kitasato University School of Medicine, 1-15-1 Kitasato, Minami-ku, Sagamihara-shi, Kanagawa, 252-0374, Japan; Department of Cardiovascular Surgery, Kochi Medical School, Kochi University, Kohasu, Oko-cho, Nankoku-shi, Kochi, 783-8505, Japan; Department of Cardiovascular Surgery, Kitasato University School of Medicine, 1-15-1 Kitasato, Minami-ku, Sagamihara-shi, Kanagawa, 252-0374, Japan; Department of Cardiovascular Surgery, Yokohama Rosai Hospital, 3211 Kozukue-cho, Kohoku-ku, Yokohama-shi, Kanagawa, 222-0036, Japan; Department of Cardiovascular Surgery, NTT Medical Center, 5-9-22 Higashi-Gotanda, Shinagawa-ku, Tokyo, 141-8625, Japan; Department of Cardiovascular Surgery, National Center for Global Health and Medicine, 1-21-1 Toyama, Shinjuku-ku, Tokyo, 162-8655, Japan; Department of Cardiovascular Surgery, National Center for Global Health and Medicine, 1-21-1 Toyama, Shinjuku-ku, Tokyo, 162-8655, Japan; Department of Cardiovascular Surgery, Kitasato University School of Medicine, 1-15-1 Kitasato, Minami-ku, Sagamihara-shi, Kanagawa, 252-0374, Japan

**Keywords:** Type A intramural haematoma, medical management, haematoma thickness, disease progression, complication

## Abstract

**Objectives:**

This study aimed to investigate changes in haematoma thickness (HT) and the maximum ascending aortic diameter (MAD) early after onset and their association with in-hospital disease progression in type A intramural haematoma (IMH).

**Methods:**

Medical records and serial computed tomography angiography (CTA) scans of 140 patients treated from April 2011 to June 2023 at 6 hospitals in Japan were retrospectively analysed. Remodelling rates (mm/h) for HT and MAD were calculated using the first 2 CTAs divided by the scan-interval time. Disease progression during hospitalization was defined as new ulcer-like projections, haematoma enlargement, false lumen aortic recanalization of the ascending aorta, or aortic rupture. Associations within these parameters and in-hospital disease progression were evaluated.

**Results:**

The mean MAD was 44.2 ± 4.9 mm, and HT was 8.1 ± 4.1 mm. In-hospital disease progression occurred in 35/140 patients (25%), and overall in-hospital mortality was 12/140 (9%). Aortic remodelling was analysed in 129/140 patients. Both HT and MAD demonstrated rapid early negative remodelling and were significantly associated with time from onset, whereas maximum values of HT and MAD within 24 hours indicated no significant correlation with time from onset. Logistic regression revealed that HT and its remodelling rate did not predict in-hospital disease progression; only the MAD remodelling rate was an independent predictor (OR, 0.71 per 0.1 mm/h, 95% CI, 0.52-0.94; *P* = .025).

**Conclusions:**

HT may have limited utility as a single-time-point imaging marker for early risk stratification, and repeated early morphological assessment may be useful in selected medically managed patients with type A IMH.

## Graphical abstract

**Figure:**
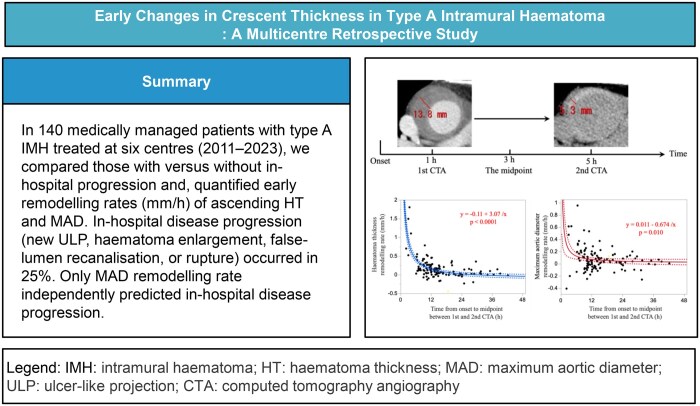

## INTRODUCTION

Acute type A intramural haematoma (IMH) is characterized by haemorrhage within the aortic media without an intimal tear.^1^^,^^2^ IMH may evolve into a life-threatening condition by progressing to classical aortic dissection, necessitating timely diagnosis and appropriate treatment.^3^ The 2024 European Society of Cardiology (ESC)/European Association for Cardio-Thoracic Surgery (EACTS) guidelines recommend emergency or urgent surgical aortic repair.^1^^,^^2^ High-risk imaging features include a maximum ascending aorta diameter (MAD) of >45–50 mm, haematoma thickness (HT) of ≥10 mm, enlarging HT, focal intimal disruption with ulcer-like projections (ULPs) and pericardial effusion at admission.^2^ A “wait-and-see strategy” has been indicated for patients at high surgical risk or with pure type A IMH without high-risk imaging features.^2^ Conversely, multiple Asian institutions have reported favourable outcomes with initial medical management under strict imaging surveillance.^1^^,^^4–8^ Historically, HT has been proposed as a predictor of adverse events, and the 2024 ESC guidelines continue to list HT >10 mm as a high-risk feature.^2^^,^^9^^,^^10^ However, HT can decrease rapidly during the acute phase, limiting the prognostic value of single-time-point assessment.^11^ Therefore, this study was expanded to a multicentre population, focusing on investigating the association between the dynamic changes in HT during disease onset and in-hospital disease progression.

## MATERIALS AND METHODS

### Ethics statement

This multicentre retrospective study at 6 cardiovascular centres in Japan was approved by the Kitasato University School of Medicine IRB (B23-093; December 4, 2023); written informed consent was waived. No data or biological material were collected for biobanking or stored for multiple and indefinite future use beyond the present study.

### Definition and diagnosis of intramural haematoma

Type A IMH was defined as crescentic/circumferential aortic wall haemorrhage without contrast opacification of a false lumen or an identifiable intimal tear on contrast-enhanced computed tomography angiography (CTA).

ULPs <15 mm in vertical length were included, whereas intramural blood pools without visible communication with the aortic lumen were not categorized as ULPs. CTA was performed using 64-row or higher multidetector CT scanners. ULP locations were classified according to the aortic landing zone.^12^ Repeat CTA was performed for transferred patients only when diagnostic uncertainty existed.

### Study population and treatment strategy

**Figure 1.**
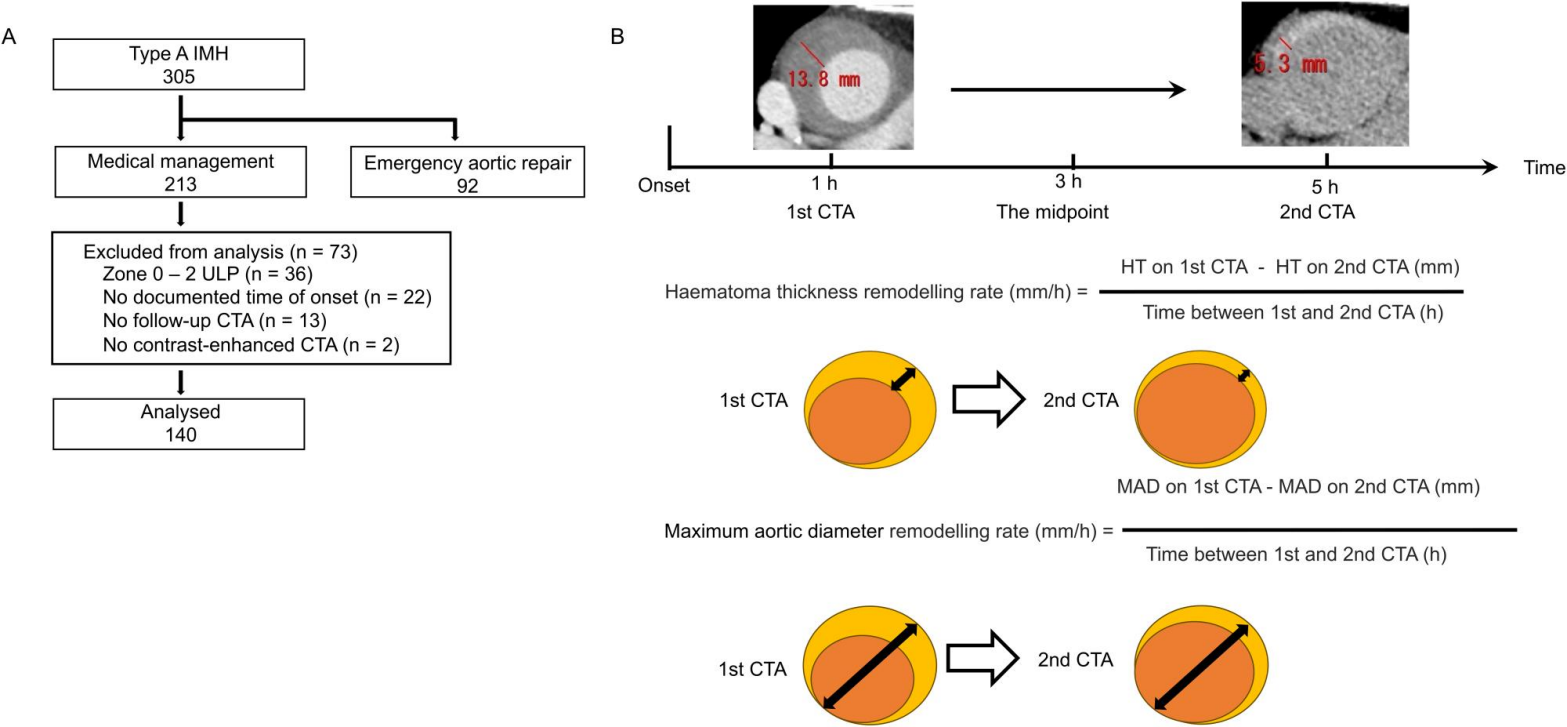
(A) Consort Diagram. Breakdown of Patients With Type A Intramural Haematoma. (B) Remodelling Rate: Definition and Calculations. Type A Intramural Haematoma Demonstrates Rapid Morphological Changes in the Early Stages of Onset. The Remodelling Rates for Haematoma Thickness and Maximum Ascending Aortic Diameter Were Calculated According to the Formula

Figure 1 illustrates the patient consort diagram. The present analysis included patients who received initial medical treatment and underwent at least 2 contrast-enhanced CTA examinations during hospitalization. Thus, this study focused on a selected cohort in which initial conservative management was selected, and serial CTA surveillance was available. After exclusions (*n* = 73), 140 patients were included. Medical management, with or without pericardial drainage, was generally indicated in patients with MAD not exceeding 50 mm, maximal HT of 11 mm or less and well-controlled pain (numerical rating scale^13^ ≤ 3/10). In addition, medical management was permitted in cases of advanced age, prohibitive surgical risk, or refusal of surgery by the patient or family. Emergency or urgent surgery was considered for patients who developed aortic rupture, false lumen canalization of the ascending aorta, progressive HT enlargement or persistent chest or back pain despite optimal blood pressure and heart rate control. The attending cardiovascular surgeons made the final decisions at each institution.

### Medical management and imaging protocol

Medical management followed institutional protocols as previously reported,^14^^,^^15^ including intensive blood-pressure control (target systolic <110 mmHg) and serial CTA surveillance. CTA was typically performed on hospital days 1, 3, 7, and 14, with additional imaging triggered by clinical change. The observation period from initial presentation to the last available clinical follow-up was calculated and is reported in the Results section.

### Morphological measurements and remodelling rate

HT and MAD were measured on multiplanar reconstruction images.^16^ The remodelling rates (mm/h) for HT and MAD were calculated employing the formula shown in Figure 1B.

### Study outcomes

The primary outcome was in-hospital disease progression; secondary outcomes included early HT/MAD remodelling rates (first 2 CTAs), maximum HT/MAD within 24 h, in-hospital mortality, and conversion to surgery. Comparisons between initial medical management and emergency surgery were not performed, as treatment allocation was non-random and outside the scope of this observational analysis.

### Definition of disease progression

In-hospital disease progression was defined as new ULP in zones 0–2, HT enlargement >2 mm, false-lumen recanalization, or aortic rupture, reflecting clinically guideline-relevant deterioration patterns. The >2 mm threshold was applied as an operational criterion to represent a change beyond expected measurement variability on serial CT.^1^^,^^3^^,^^16^

### Statistical analysis

Continuous variables are presented as mean ± SD or median (IQR), and categorical variables as *n* (%). Between-group comparisons used the Mann-Whitney *U* test for continuous variables and Fisher’s exact test or χ^2^ test for categorical variables. Distributional assumptions were assessed primarily by graphical inspection (histograms/box plots), with the Shapiro-Wilk test used as a supplementary check; given non-normal distributions and small subgroup sizes, non-parametric tests were applied as the default for continuous variables. Relationships between imaging timing and morphological indices (remodelling rates and maximum HT/MAD within 24 hours) were assessed using regression models; candidate functional forms (linear, log-transformed, inverse [1/*x*]) were compared by AICc, selecting the lowest AICc (log-transformed was chosen when ΔAICc <2). Robust regression (M-estimation) was performed as sensitivity analysis (**Table S1**). Predictors of in-hospital disease progression were evaluated using univariable and multivariable logistic regression. Discriminative ability and optimal cutoffs were assessed using ROC analysis with Youden’s index. In addition, clinically meaningful cut-offs were evaluated as sensitivity analyses to support interpretability and assess potential non-linearity (**Table S5**). Complete-case analyses for each model (no imputation) were conducted to handle missing data; therefore, the number of observations differed by analysis. A two-sided *P* < .05 was considered significant. All analyses were performed using JMP Pro 18 (SAS Institute Inc.). Mixed-effects modelling was not applied because the primary objective used patient-level early summary changes (remodelling rates) derived from serial CTA with heterogeneous timing across centres. Prospective studies with standardized CTA schedules are needed to enable formal longitudinal mixed-effects modelling. Odds ratios, 95% CIs, and *P* values are reported with standardized decimal precision across tables to avoid overstating numerical precision.

## RESULTS

### Patient characteristics and outcomes

**Figure 2.**
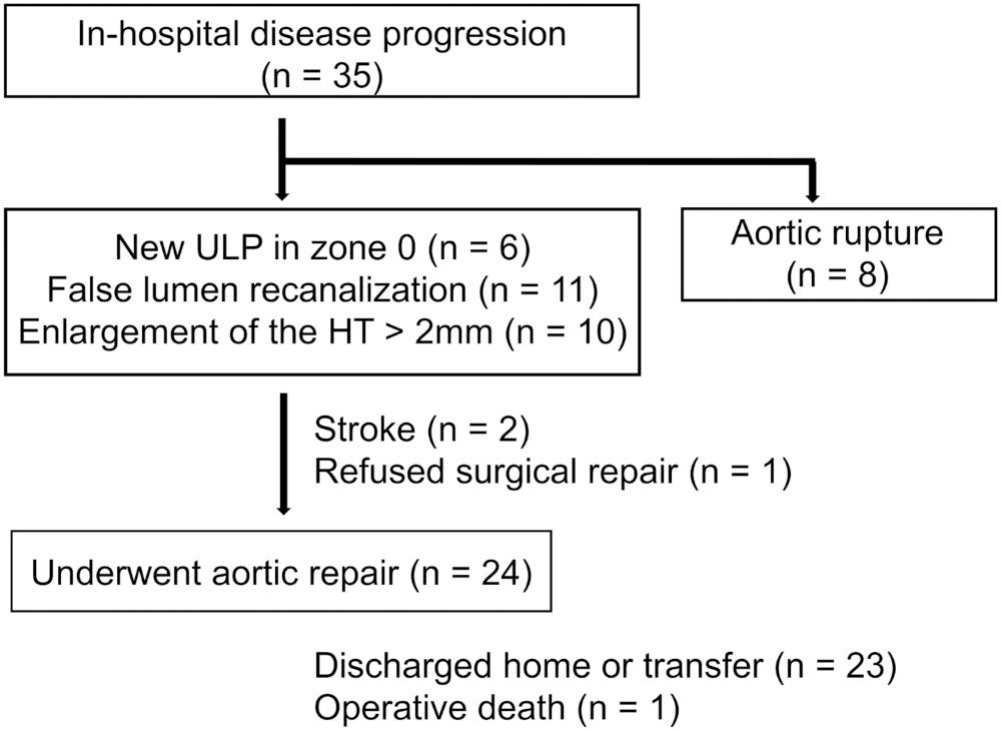
Outcomes After in-Hospital Disease Progression. Flow Diagram Showing the Components of In-Hospital Disease Progression and Subsequent Clinical Course

The median observation period from initial presentation to the last available clinical follow-up was 1063 days (IQR, 197-2046; range, 1-4572). Table 1 summarizes the baseline characteristics and clinical outcomes of the 140 patients in this study. A ULP was absent in 84 patients (60%); when present, it was located in zone 3 in 11 (8%), zone 4 in 36 (26%), and zone 5 or beyond in 9 (6%) patients. The mean initial MAD and HT were 44.2 ± 4.9 mm and 8.1 ± 4.1 mm, respectively. Pericardial effusion at admission was observed in 64 patients (46%), and pericardial drainage was performed in 21 patients (15%). Table 2 presents baseline characteristics. Baseline MAD and HT were comparable between groups, whereas in-hospital mortality was higher in the progression group. Figure 2 summarizes progression and subsequent clinical course. In-hospital disease progression occurred in 35 patients (25%). Progression comprised new ULP formation (*n* = 6), false lumen recanalization of the ascending aorta (*n* = 11), HT enlargement >2 mm (*n* = 10) or aortic rupture (*n* = 8). Thus, the composite end-point included both imaging-defined morphological progression (new ULP, recanalization or HT enlargement) and major clinical events (rupture). Among the 27 patients with progression other than rupture, 24 underwent aortic repair during the initial hospitalization, with 23 survivors discharged. Three patients did not undergo surgery (stroke, *n* = 2; surgical refusal, *n* = 1). Overall, in-hospital mortality was reported in 12 patients (9%), comprising 8 ruptures, 1 operative death and 3 non-operative deaths due to severe stroke. Thus, 11 patients died without undergoing surgical repair (8 from rupture and 3 from severe stroke). Among the 35 patients with disease progression, 24 (69%) proceeded to aortic repair after progression, whereas 11 did not (rupture, *n* = 8; stroke, *n* = 2; post-progression refusal, *n* = 1). Among patients who died of rupture during a watch-and-wait strategy, one had undergone pericardial drainage and subsequently died from rupture 3 days later. Among the 35 patients with disease progression, the initial MAD (44.7 ± 4.8 mm) and HT (8.5 ± 3.3 mm) were comparable to those of another study.^11^^,^^15^ The median time to disease progression was 6.5 days (IQR, 4-17). The HT measured immediately before progression demonstrated no meaningful reduction from baseline. Notably, the initial HT in patients with fatal rupture (7.7 ± 3.7 mm) was similar to that of the entire cohort. Among the 140 medically managed patients, 12 (8.6%) had an initial MAD >50 mm (**Table S4**). Initial non-operative management in this subgroup was chosen because of refusal of surgery at presentation (*n* = 4), advanced age (*n* = 3), rapid early MAD regression on repeat CTA within 6 h (*n* = 1), post-cardiopulmonary arrest resuscitation (*n* = 3), or unknown reasons (*n* = 1). Pericardial effusion was present in 8/12 patients and pericardial drainage was performed in 2/12. Two patients died of aortic rupture during hospitalization: one who refused surgery at presentation and underwent pericardial drainage, and one managed conservatively because of advanced age.

**Table 1.** Patient Demographics (*n* = 140)

Age (years), mean ± SD	71 ± 12
Female sex, *n* (%)	78 (56)
Cardiopulmonary arrest on admission, *n* (%)	2 (1)
Loss of consciousness, *n* (%)	38 (27)
Paralysis/paresis, *n* (%)	8 (6)
Pericardial effusion, *n* (%)	64 (46)
Hypertension, *n* (%)	101 (72)
Hyperlipidemia, *n* (%)	29 (21)
Diabetes mellitus, *n* (%)	11 (8)
Ischaemic heart disease, *n* (%)	2 (1)
Hemodialysis, *n* (%)	3 (2)
Steroid use, *n* (%)	6 (4)
Location of ULP, *n* (%)	
None	85 (61)
Zone 3	11 (8)
Zone 4	36 (26)
Zone 5	5 (4)
Zone 6	1 (1)
Zone 7	1 (1)
Zone 8	1 (1)
Time from onset to the 1st CTA (h), median (IQR)	2.7 (IQR, 1.4-4.6)
MAD on the 1st CTA (mm), mean ± SD	44.2 ± 4.9
Ascending aorta HT on the 1st CTA (mm), mean ± SD	8.1 ± 4.1
In-hospital disease progression, *n* (%)	35 (25)
New ULP in zone 0, *n*	6
False lumen recanalization, *n*	11
Enlargement of the HT > 2 mm, *n*	10
Aortic rupture, *n*	8
Crossover to surgical aortic repair, *n* (%)	24 (17)
In-hospital mortality, *n* (%)	12 (9)

Data are presented as mean ± SD or *n* (%). Continuous variables are reported as mean ± SD for clinical familiarity, although some distributions were non-normal. Therefore, group comparisons used non-parametric tests as described in the “Methods” section.

Abbreviations: CTA, computed tomography angiography; HT, haematoma thickness; MAD, maximum ascending aortic diameter; ULP, ulcer-like projection.

**Table 2.** Baseline Characteristics and in-Hospital Outcomes Stratified by in-Hospital Disease Progression

Variable	No progression (*n* = 105)	In-hospital disease progression (*n* = 35)	*P* value
Age (years), mean ± SD	71 ± 12	73 ± 10	.356
Female sex, *n* (%)	57 (54)	21 (60)	.612
Hypertension, *n* (%)	81 (79)	20 (57)	.336
Hyperlipidemia, *n* (%)	24 (23)	5 (14)	.368
Diabetes mellitus, *n* (%)	10 (10)	1 (3)	.198
Ischaemic heart disease, *n* (%)	0	2 (6)	.010
Hemodialysis, *n* (%)	2 (2)	1 (3)	.938
Steroid use, *n* (%)	4 (4)	2 (6)	.857
Pericardial effusion, *n* (%)	45 (43)	19 (54)	.308
Maximum ascending aorta diameter on the 1st CTA (mm), mean ± SD	43.9 ± 5.1	45.1 ± 4.4	.225
Ascending aorta haematoma thickness on the 1st CTA (mm), mean ± SD	8.0 ± 4.3	8.6 ± 3.4	.432
In-hospital mortality, *n* (%)	3 (3)	9 (26)	<.001

Values are reported as mean ± SD or *n* (%). Between-group comparisons used the Mann-Whitney *U* test for continuous variables and Fisher’s exact test for categorical variables. Tests were two-sided, with *P* < .05 considered statistically significant.

Abbreviation: CTA, computed tomography angiography.

### HT and maximal aortic diameter in the ascending aorta: remodelling rate over time

**Figure 3.**
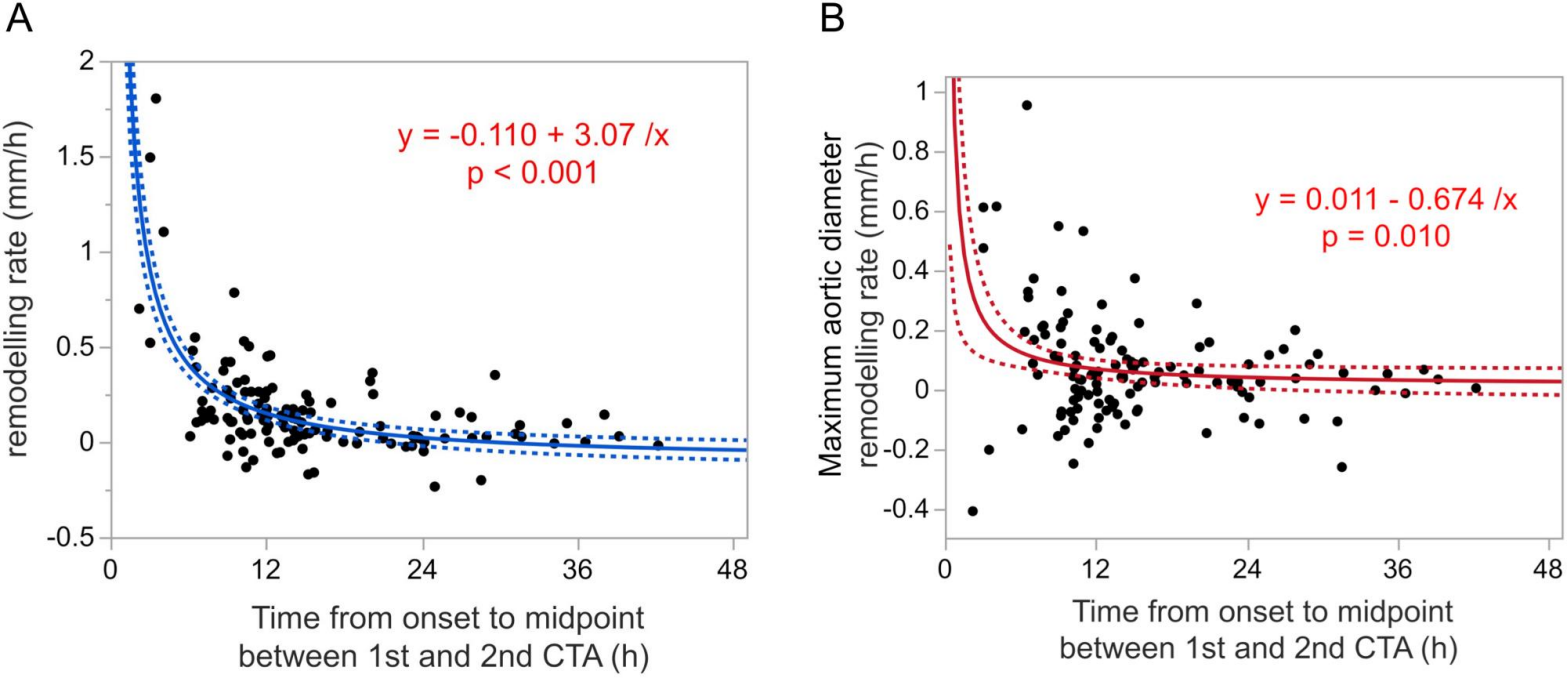
(A) Haematoma Thickness Remodelling Rate. The Haematoma Thickness Remodelling Rate Versus Midpoint Time from Onset Between the First and Second Computed Tomography Angiography (CTA) (*n* = 129). A Reciprocal Regression Model Demonstrated a Significant Association (*y* = –0.11 + 3.07/*x*; *P* < .001). Dotted lines Denote the 95% CI. (B) Maximum Ascending Aortic Diameter Remodelling Rate. Remodelling Rate of the Maximum Ascending Aortic Diameter Versus the Time From Onset to the Midpoint Between the First Two CTAs (*n* = 129). A Reciprocal Regression Model Showed a Significant Association (*y* = 0.011 – 0.674/*x*; *P* = .010). Dotted Lines Indicate the 95% CI

Of the 140 patients, 11 were excluded from early remodelling analysis (rupture before second CTA, *n* = 1; midpoint >48 h, *n* = 10); thus, 129 patients were analysed (Figure 3). HT and MAD remodelling rates showed an inverse reciprocal association with time from onset to the midpoint between the first two CTAs (HT, *P* < .001; MAD, *P* = .010). These results reveal that morphological remodelling of type A IMH is most dynamic within the first hours after onset and rapidly declines. A sensitivity analysis that included the 11 patients with delayed imaging did not change the regression results (data not shown).

### Maximum values within 24 hours of HT and MAD in the ascending aorta

**Figure 4.**
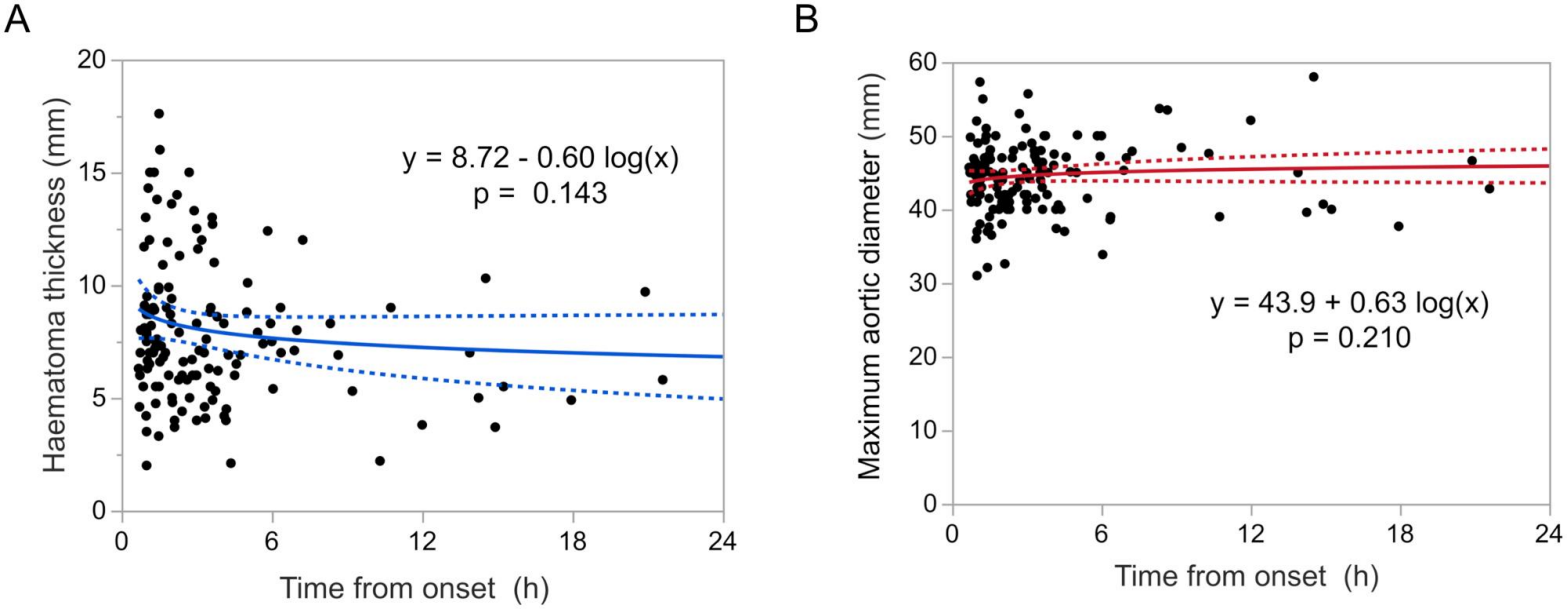
(A) Maximum Haematoma Thickness Within 24 hours After Onset. The Maximum Haematoma Thickness (HT) Measured Within 24 hours from Onset (*n* = 137). A Logarithmic Regression Model Demonstrated No Significant Association. Dotted Lines Denote the 95% CI. (B) Maximum Ascending Aortic Diameter Within 24 Hours After Onset. The Maximum Ascending Aortic Diameter (MAD) Measured Within 24 hours From Onset (*n* = 137). A Logarithmic Regression Model Demonstrated No Significant Association. Dotted Lines Denote the 95% CI

The association between time from onset and maximum HT and MAD within 24 hours was analysed in 137 patients (Figure 4); three patients were excluded because the first CTA was performed >24 hours after onset. Logarithmic regression showed no significant correlation, and no distinct temporal trend was identified within 24 hours.

### Predictors of in-hospital disease progression

MAD remodelling rate was associated with in-hospital disease progression in univariable analysis (OR 0.74 per 0.1 mm/h, 95% CI, 0.54-0.98; *P* = .034) and remained the only independent predictor in the multivariable model including age and HT remodelling rate (OR 0.71 per 0.1 mm/h, 95% CI 0.52-0.94; *P* = .025) (Table 3). The MAD remodelling rate can take negative values when MAD decreases; thus, an OR of <1 indicates that greater early MAD regression (more negative remodelling) is associated with lower odds of in-hospital disease progression, whereas lack of regression or early expansion is associated with higher odds. Neither age nor HT remodelling rate reached statistical significance, indicating that early dynamic shrinkage of the ascending aorta has greater prognostic relevance than HT alone. Confidence intervals were relatively wide, and the magnitude of the associations should be interpreted with appropriate caution, considering the limited number of progression events. Further, the discriminative ability of baseline HT for in-hospital disease progression was limited (AUC, 0.59; **Table S3**).

**Table 3.** Univariable and Multivariable Logistic Regression Analyses for Predictors of in-Hospital Disease Progression

Variables	Univariate OR (95% CI)	*P* value	Multivariate OR (95% CI)	*P* value
Age (year)	1.0 (1.0-1.1)	.342	1.0 (1.0-1.1)	.431
Male sex	0.8 (0.4-1.8)	.648		
Loss of consciousness	1.4 (0.6-3.8)	.502		
Pericardial effusion	1.3 (0.8-2.1)	.267		
No ulcer-like projection	1.4 (0.6-3.2)	.482		
HT remodelling rate (per 0.1 mm/h)	1.1 (1.0-1.3)	.188	1.1 (0.9-1.3)	.557
MAD remodelling rate (per 0.1 mm/h)	0.7 (0.5-1.0)	.034	0.7 (0.5-0.9)	.025
Maximum HT within 24 h	1.1 (1.0-1.2)	.146		
Maximum MAD within 24 h	1.1 (1.0-1.2)	.062		

ORs for remodelling rates are expressed per 0.1 mm/h (ie, remodelling rate variables were multiplied by 10) to improve interpretability. Odds ratios and 95% CIs are generally rounded to one decimal place to avoid overstating precision.

Abbreviations: CI, confidence interval; HT, haematoma thickness; MAD, maximal ascending aortic diameter.

## DISCUSSION

This multicentre retrospective study assessed the early morphological behaviour of type A IMH using serial CTA, focusing on the drastic changes in HT and MAD. This study reveals 3 major observations. First, both HT and MAD undergo rapid and substantial remodelling during the early hours following onset. Second, the maximum HT and MAD values within 24 hours were not associated with the time from onset. Third, among the dynamic morphological variables, only the MAD remodelling rate independently predicted in-hospital disease progression, whereas the HT remodelling rate was not significantly related to in-hospital disease progression. Specifically, greater early MAD regression was associated with a lower likelihood of in-hospital disease progression. These findings challenge the reliability of static thresholds—particularly the guideline-endorsed 10-11 mm HT criterion—as indicators of early risk in type A IMH.

Previous studies have indicated that HT is associated with complications in type A IMH with medical management.^4^^,^^10^ Several major guidelines still cite HT of ≥10-11 mm as a high-risk imaging finding.^1^^,^^2^ However, studies supporting this threshold are often small cohorts, with unclear reasons for setting the 10 mm criteria.^1^^,^^3^ Furthermore, these studies appeared insufficient to support the guidelines, as the presence or location of ULP, nor the time course changes of HT, were not considered.^10^ In this study, the mean initial HT was 8.1 ± 4.1 mm and the maximum HT within 24 h remained comparable. Notably, even among the 8 patients who died from aortic rupture during hospitalization, the mean HT was only 7.7 ± 3.7 mm (median time to rupture: 3.5 days), significantly lower than the thresholds recommended by the current guidelines. HT demonstrated a nonlinear and time-dependent decrease within the first 1–2 days after onset (Figure 3), indicating that it was a dynamic parameter affected by imaging timing. The discriminative ability of HT for predicting in-hospital disease progression was poor, with an area under the curve of 0.59 in the ROC analysis (**Table S3**). These findings suggest that single-time-point HT is unreliable for early risk stratification, supporting serial morphological assessments. Conversely, the MAD remodelling rate was associated with in-hospital disease progression and remained the only independent predictor in the multivariate analysis. The results indicated that with decreased MAD on the second CTA scan, disease progression was less expected; however, continued careful observation was necessary when MAD remained unchanged. These findings support the need for continued morphological reassessment during an initial watch-and-wait strategy, although the optimal timing and frequency of CTA remain to be established. In our real-world cohort, conservative management was preferentially selected for patients with prohibitive operative risk or for those who refused surgery. A similar selective strategy, including pericardial drainage without immediate aortic repair in carefully chosen patients with type A intramural haematoma complicated by tamponade, has also been reported.^8^ A small number of patients with initial MAD of >50 mm was treated medically for these reasons or because of rapid early MAD regression on repeat CTA. These implications should be interpreted as hypothesis-generating and primarily applicable to patients selected for an initial watch-and-wait strategy, given the retrospective design and end-point heterogeneity. Future studies are warranted to develop a time-dependent risk prediction model, involving standardized CTA timing in a prospective, multicentre registry study. Several limitations of this study should be acknowledged. First, this multicentre study included data from 6 institutions; however, its retrospective design may have introduced selection and imaging timing bias, as the cohort was limited to patients initially treated conservatively who underwent serial CTA. Further, despite efforts to standardize the timing of image analysis, heterogeneity in CT angiography acquisition protocols across participating centres may have affected quantitative measurements. Second, the remodelling rate and the midpoint time lack mathematical independence, because both variables partially incorporate the CT scan interval. The effect of scan timing on the observed temporal patterns cannot be completely excluded. Third, imaging frequency may affect the analysis of maximum HT and MAD values within 24 hours, because patients undergoing more frequent CT examinations are more likely to have higher maximum values detected. Fourth, some potentially relevant clinical variables (eg, pain severity, blood pressure control) were not uniformly captured across all centres and were therefore excluded from the present analyses. Imaging changes may have influenced subsequent management decisions, potentially affecting observed outcomes. Fifth, our composite definition of in-hospital disease progression includes events with different clinical weight and pathophysiological meaning (eg, new ULP formation, aortic recanalization, HT enlargement, rupture). Considering the heterogeneity of the composite end-point, our results are primarily interpreted at the composite level; component-specific implications (eg, rupture vs non-rupture progression) should be considered exploratory. Finally, this study population comprised exclusively of Japanese patients, which may limit the generalisability of the findings to other racial or regional populations. However, the multicentre design strengthens our findings compared with single-centre analyses and supports dynamic morphological assessment in type A IMH.

## CONCLUSIONS

In patients with type A IMH indicated for medical management, HT demonstrated marked early regression and poor prognostic utility when assessed at a single time point. The HT remodelling rate was not associated with in-hospital disease progression, whereas the MAD remodelling rate was the only independent prognostic predictor. These findings suggest that static HT may be insufficient for early risk stratification in this selected cohort. These findings support continued morphological reassessment during initial medical management, although the optimal timing and frequency of imaging remain to be established.

## Supplementary Material

ivag146_Supplementary_Data

## Data Availability

The data underlying this article cannot be shared publicly due to the privacy of individuals who participated in the study. The data will be shared on reasonable request to the corresponding author.

## Data Availability

The datasets generated and/or analysed during the current study are stored in the secure institutional database of Kitasato University. Data will be made available upon reasonable request to the corresponding author, in accordance with the institution’s data-sharing policies and approval process.

## ABBREVIATIONS

CTA: computed tomography angiography
HT: haematoma thickness
IMH: intramural haematoma
MAD: maximum ascending aorta diameter
ULP: ulcer-like projection
