# Supplementary material for: Early Dynamic Changes in Haematoma Thickness in Medically Managed Type A Intramural Haematoma: A Multicentre Retrospective Study
Source: Interdiscip Cardiovasc Thorac Surg. 2026 Jun 8;41(6):ivag146. doi: 10.1093/icvts/ivag146 (PMC13275119; doi:10.1093/icvts/ivag146)
Supplement: ivag146_Supplementary_Data [file ivag146_supplementary_data.docx]

Supplementary Table S1. Model selection for time-dependent curve fitting using AICc (Figures 3 and 4)

|  | Model | AICc | Selected (Yes/No) |
| --- | --- | --- | --- |
| Figure 3A | 1/x | -41.5 | Yes |
|  | log(x) | -12.7 | No |
| Figure 3B | 1/x | -77.8 | No |
|  | log(x) | -81.6 | Yes |
| Figure 4A | log(x) | 670.0 | Yes |
|  | linear | 670.5 | No |
| Figure 4B | log(x) | 787.8 | Yes |
|  | linear | 788.3 | No |

The best-fitting functional form was selected based on the Akaike Information Criterion corrected for small sample size (AICc), with lower values indicating better fit. When the difference between candidate models was small (ΔAICc < 2), the log-transformed model was selected for consistency with other analyses.

Supplementary Table S2. Robust regression (M-estimation) sensitivity analyses for Figures 3 and 4

|  | Sigma (≈RMSE) | p value | Intercept (Robust est.) | SE (Intercept) | Slope (Robust est.) |
| --- | --- | --- | --- | --- | --- |
| Figure 3A | 0.17 | <0.001 | 0.257 | 0.037 | -0.007 |
| Figure 3B | 0.15 | 0.007 | 0.115 | 0.031 | -0.003 |
| Figure 4A | 3.16 | 0.469 | 8.069 | 0.392 | -0.044 |
| Figure 4B | 4.86 | 0.362 | 44.16 | 0.511 | 0.065 |

Robust regression (M-estimation) was performed as a sensitivity analysis to reduce the influence of potential outliers in the bivariate relationships shown in Figures 3 and 4. Sigma approximates the root mean squared error (RMSE).

Supplementary Table S3. ROC analysis of morphological parameters within 24 h after onset for predicting in-hospital disease progression

|  | Cut-off (mm) | Predicted probability at cut-off | Sensitivity | Specificity | Youden | AUC |
| --- | --- | --- | --- | --- | --- | --- |
| Haematoma thickness | 8.3 | 0.23 | 0.59 | 0.68 | 0.27 | 0.59 |
| Maximum ascending aortic diameter | 45.1 | 0.24 | 0.63 | 0.61 | 0.24 | 0.61 |

Receiver operating characteristic (ROC) analysis assessed the discriminative ability of morphological parameters measured within 24 h after onset for predicting in-hospital disease progression (positive outcome). The optimal cut-off was defined by maximising Youden’s index.

Supplementary Table S4. Case-level details of medically managed patients with an initial MAD >50 mm (n=12)

| Case No. | Age (years) | Sex | ULP location | 1st MAD (mm) | 1st HT (mm) | 2nd MAD (mm) | 2nd HT (mm) | Key reason for initial non-operative management | Pericardial effusion | In-hospital outcome |
| --- | --- | --- | --- | --- | --- | --- | --- | --- | --- | --- |
| 1 | 81 | F | None | 53.5 | 6.9 | 53.4 | 6.3 | Refusal at presentation | No | Survived |
| 2 | 80 | F | None | 57.3 | 6.5 | 53.4 | 3.2 | Refusal at presentation | Yes (drained) | Survived |
| 3 | 67 | F | None | 55.7 | 11.6 | 49.1 | 7.8 | Rapid early regression | Yes (undrained) | Survived |
| 4 | 75 | F | None | 52.1 | 3.8 | 50.1 | 5 | Refusal at presentation | Yes (drained) | Died of rupture |
| 5 | 87 | F | None | 56.2 | 7.4 | 60.7 | 4.9 | Advanced age | Yes (undrained) | Survived |
| 6 | 85 | F | None | 52 | 15 | 53 | 12 | Advanced age | Yes (undrained) | Died of rupture |
| 7 | 74 | M | Zone 4 | 51 | 6 | 50 | 7 | Refusal at presentation | No | Survived |
| 8 | 74 | M | None | 55 | 39 | 52 | 40 | Post-CPA | Yes (undrained) | Survived |
| 9 | 86 | M | None | 52 | 4.2 | 52.1 | 3.4 | Post-CPA | No | Survived |
| 10 | 87 | F | None | 58 | 10.3 | 57 | 14 | Advanced age | Yes (undrained) | Survived |
| 11 | 76 | M | Zone 4 | 51 | 5.5 | 50 | 2.3 | Post-CPA | Yes (undrained) | Survived |
| 12 | 67 | M | Zone 4 | 51 | 12.4 | 50 | 11 | Unknown | No | Survived |

Values are shown in mm unless otherwise stated. “Yes—drainage” indicates pericardial effusion requiring pericardial drainage due to clinical tamponade during the index hospitalisation.

Abbreviations: MAD, maximal ascending aortic diameter; HT, haematoma thickness; ULP, ulcer-like projection; CPA, cardiopulmonary arrest; CTA, computed tomography angiography.

Supplementary Table S5. Sensitivity analyses using clinically meaningful cut-offs for predictors of in-hospital disease progression

| Predictor (category) | N | Univariable OR (95% CI) | p value | Multivariable OR (95% CI) | P value |
| --- | --- | --- | --- | --- | --- |
| Initial MAD on first CTA: 46–50 mm vs ≤45 mm | 117 | 1.8 (0.7–4.4) | 0.208 |  | |
| Initial MAD on first CTA: >50 mm vs ≤45 mm | 93 | 2.0 (0.5–7.4) | 0.307 |  | |
| Initial HT on first CTA: ≥10 mm vs <10 mm | 129 | 1.8 (0.7–4.5) | 0.215 | 2.0 (0.7–5.4) | 0.166 |
|  |  |  |  |  |  |
| MAD remodelling: non-regression (≥0) vs regression (<0) | 129 | 3.0 (1.3–6.9) | 0.011 | 2.9 (1.2–6.7) | 0.016 |
|  |  |  |  |  |  |
|  |  |  |  |  |  |

Odds ratios (ORs) are reported with 95% confidence intervals (CI). These categorical analyses were prespecified as sensitivity analyses to assess potential non-linearity and improve clinical interpretability. N varies across analyses because of missing values in covariates used for each model. Multivariable estimates are shown only for the prespecified categorical sensitivity model including age (continuous), initial HT (≥10 vs <10), and MAD remodelling category (non-regression [≥0] vs regression [<0]) (complete-case analysis, N=129).

Abbreviations: CTA, computed tomography angiography; MAD, maximal ascending aortic diameter; HT, haematoma thickness; CI, confidence interval.

Supplementary Table S6. Timing-adjusted multivariable logistic regression for in-hospital disease progression

| Covariate | Adjusted OR (95% CI) | p value |
| --- | --- | --- |
| Age (year) | 1.0 (1.0–1.1) | 0.385 |
| HT remodelling rate (per 0.1 mm/h) | 1.0 (0.9–1.2) | 0.676 |
| MAD remodelling rate (per 0.1 mm/h) | 0.7 (0.5–0.9) | 0.023 |
| Onset-to-midpoint time (per hour) | 0.99 (0.95–1.02) | 0.599 |

ORs for remodelling rates are expressed per 0.1 mm/h (i.e., remodelling rate variables were multiplied by 10). Midpoint time indicates the time from onset to the midpoint between the first two CTAs (complete-case analysis, N=129). Odds ratios and 95% confidence intervals are generally rounded to one decimal place to avoid overstating precision; however, the onset-to-midpoint time estimate is shown to two decimal places because rounding to one decimal place would obscure interpretability.

Abbreviations: CI, confidence interval; CTA, computed tomography angiography; HT, haematoma thickness; MAD, maximum ascending aortic diameter.
